# The Symptomatology of Early Ectopic Gestation

**Published:** 1900-08

**Authors:** Maud J. Frye

**Affiliations:** Buffalo, N. Y.


### THE SYMPTOMATOLOGY OF EARLY ECTOPIC GESTATION.

BY MAUD J. FRYE, M. D., Buffalo, N. Y.

I WAS fortunate enough not long ago to see in my own practice two cases of early ectopic gestation occurring within a few weeks of each other. I should not venture to offer to this association a paper founded on such a limited personal experience of a condition no longer regarded as rare, but for the contrast in symptoms which the patients presented both before and after rupture. With the belief that on this account a report of these cases will be interesting, if not instructive, I present such report to you:

Mrs. A., married, aged 23, mother of one child, about two years of age, first came under my care February 8, 1900. She was a slender, delicate, anemic woman. Her general history was fair, except for an illness the previous fall, diagnosticated inflammation of the bowels by her attending physician. Her menstruation was usually regular. She had missed the period due January 26th and was suffering with pain, such as accompanies early abortion. Examination showed a retroverted, somewhat enlarged uterus, the os patulous, the cervix not so soft as in normal pregnancy. No tubal swelling was

1. Read at the twenty-fifth annual meeting of the Alumni Association of the Medical Department of the University of Buffalo, April 27, 1900.

discovered, possibly because I believed that I was dealing with a normal uterine pregnancy and did not look for any. Long years of experience are necessary to overcome the tendency to find and diagnosticate the condition one has in mind—an illustration of the influence mind has over matter. The patient was kept in bed and given viburnum and the pains promptly subsided. There was no discharge of blood or of decidual tissue. On February 16th, eight days later, about 8 a.m., Mrs. A. was seized with sharp and severe pains in the lower part of the abdomen, chiefly on the right side. There was also tenderness on the right side of the abdomen. The pulse was rather weak, but not frequent. The patient volunteered the information that she had had repeated similar attacks in months gone by, a statement which did not aid the diagnosis. The pain passed off and did not return until about 11 p.m. At one o'clock on the morning of the 17th, I found the patient profoundly anemic; pulse, 150, and barely perceptible, great pain in the abdomen, thirst, and the other usual symptoms of internal hemorrhage. Vaginal examination showed fulness to the right of the uterus. The diagnosis of ruptured tubal pregnancy was concurred in by Dr. Frederick and the patient was at once removed to the Woman's Hospital, where, at 4 a.m., abdominal section was done and the ruptured tube removed. The abdominal cavity was filled with blood.

Although in a precarious condition when the operation was finished the patient promptly rallied and made an uneventful recovery. It is interesting to note that the unimpregnated tube was so diseased as to be functionless and it was therefore removed. Inflammation of the bowels in the case probably meant pelvic peritonitis.

Mrs. B., aged 21, I saw for the first time February 9, 1900; she was a thin, anemic woman, married eighteen months; had never been pregnant. Shortly after marriage she suffered from an attack of pelvic peritonitis, gonorrheal in origin. She had never been well since, having attacks of pelvic pain of greater or less severity at frequent intervals. For the past few weeks she had been suffering rather more pain than usual. Her menses were regular. The whole pelvis was very tender and an examination therefore somewhat unsatisfactory. Some thickening of the structures to the left side of the uterus could be made out and a mass to the right. The uterus itself was hard and not large. A diagnosis of chronic salpingitis, possibly of tubal abscess, was made and an operation advised. On March 6th last this was done at the Woman's Hospital by Dr. Frederick.

Instead of the expected pyosalpinx a ruptured tubal pregnancy was found upon the right side, with a chronic catarrhal salpingitis on the left. The amount of blood was approximately a pint, clotted and walled off, with some fluid blood, the condition evidently the result of slow hemorrhage. The patient had presented none of the usual and expected signs of tubal pregnancy, either before or after rupture, except a long continued sero-sanguinolent flow at her last menstrual period, the significance of which was not appreciated until the abdominal cavity was opened. She had not had more pain than she had frequently suffered from in previous months, nor was it different in character. No menstrual period had recently been missed. If any shreds of decidual tissue had been passed the patient had not observed them. A most careful and repeated cross-examination of the patient after her recovery from operation failed to discover the symptoms which usually accompany tubal pregnancy before and after rupture. Only once had she thought herself pregnant, in June, 1899, when her menstruation was delayed three or four days. Her pulse frequently observed for some weeks before she entered the hospital was never above eighty.

The chief symptoms of ectopic pregnancy before rupture are usually stated as being irregular menstruation, pelvic pain, and the presence of a tumor outside the uterus, or expanded somewhat: Tubal swelling and enlargement of the uterus, associated with suppression of the menses often followed, after a brief period, by sero-sanguinolent discharges and increased flow at the menstrual period with paroxysmal pains, radiating from the side of the pelvis upon which the affected tube is situated, and with the expulsion of uterine decidua at the end of the second or in the course of the third month. These symptoms and physical signs are not always present. In the first case reported, there were no symptoms until the very day of rupture, indicative of ectopic gestation. A more thorough examination when the patient was first seen might have aroused the suspicion of extra-uterine pregnancy and led to correct diagnosis before rupture with serious hemorrhage had occurred. Yet I have seen operated upon for tubal abortion a patient whom a far abler physician than myself had, but a short time before, carefully examined under anesthesia with the possibility of extrauterine pregnancy in mind without discovering the tubal gestation, which later operation showed to have then been present.

In the case of Mrs. B., the character of the discharge was the only symptom presented which might have drawn attention to the true nature of her ailment. The American Text-book of Gynecology, says:

The condition most likely to be confused with an ectopic gestation is probably a tube distended with serum or pus, especially the latter. The physical signs of the two conditions prior to rupture often closely resemble one another. The chief point in their differentiation is the difference in their clinical history.

In this case there was no history looking toward pregnancy. Not only is it easy to fail to diagnosticate a tubal pregnancy when it is present; it is not impossible to diagnosticate a non-existent ectopic gestation. For example, Mrs. X. skipped one menstrual period, shortly after she began to have pain on the right side of the pelvis, one attack being especially severe. The uterus was slightly enlarged; there was a small swelling to the right of the uterus. Diagnosis, tubal pregnancy. Section showed a uterine pregnancy with a small cyst of the right ovary.

It is more especially to the symptoms following rather than preceding rupture, that I wish to call your attention. My first patient presented the classical and commonly accepted picture of tubal pregnancy with intraperitoneal rupture, viz.: yawning, languor, faintness, thirst, clammy perspiration, rapid pulse, intermittent vomiting, and acute anemia. The second, with a considerable amount of blood in the peritoneal cavity, had presented no symptoms at all pointing to internal hemorrhage.

Including the cases herewith reported, I have seen seventeen operations in which ruptured tubal pregnancy was found, usually when it was the condition operated for, sometimes when it had been unsuspected. In three the rupture was subperitoneal. Of the remaining fourteen, in which rupture into the peritoneal cavity had occurred, only three were emergency cases, presenting the grave symptoms of internal hemorrhage, which demand immediate surgical interference. The conclusion toward which these few cases point may be stated in the following paragraph quoted from Lusk, in Keating and Coe's Gynecology:

Even when rupture takes place the hemorrhage is not necessarily fatal. Mr. Tait insists on the relative harmlessness of most cases of hematoma, due to rupture occurring between the folds of the broad ligament; while the records of celiotomy show that, even with intraperitoneal rupture, the hemorrhage has often been moderate in amount, and did not in itself furnish the occasion for surgical interference.

224 ALLEN STREET.

DISCUSSION OF DR. FRYE'S PAPER.

Dr. C. C. FREDERICK: I take particular pleasure in discussing this paper, as I happened to see the two cases cited by Dr. Frye. I think the deductions she has drawn, and the lessons taught by the differing symptoms, and unlike conditions exhibited by these two patients are very worthy of consideration. This matter of ectopic pregnancy is a very important one, because it occurs very frequently, much oftener than we were taught to believe in the past. It used to be considered one of the rarest occurrences; now it is not at all rare. In the past few years I have several times operated on as many as three or four in one month—in fact, two or three months ago I operated on four in one week; but, of course, they are likely to appear in schools. But it is of very frequent occurrence, and consequently should be taken into consideration when a woman has pain and symptoms of internal hemorrhage. For instance, four or five years ago I was called to see a case, a woman in most serious condition, absolutely pulseless and blanched. The physician had been with her nearly all day long—over twelve hours—administering all sorts of restoratives, but had never thought of the possibility of ectopic gestation. She died before anything could be done; and after death two pailfuls of clotted blood were removed from her abdomen.

The fact stated by Dr. Frye is very pertinent and true, that a vast majority of instances of rupture are not cases for emergency operation. It is but a small proportion of cases where rupture demands an immediate operation, that is for acute anemia following the rupture; they are exceptional cases, the majority being slow bleeding, with recurrence of hemorrhage possible. It starts in, the patient becomes faint and as a result of lowering arterial tension clots form and the bleeding ceases; the patient rallies, and in a day or two, as the tension rises, there may be another hemorrhage. They may last for days or even weeks. I operated on a case two years ago where rupture had occurred about the second month. It had gone on growing subperitoneal; there had been recurrent hemorrhages until there was a tumor as large as a full term pregnancy—all subperitoneal, the whole abdominal contents lifted up by concentric layers of blood clots—and she had had a hemorrhage only a few days before, indicated by a fresh clot found. The fetus had developed until about the sixth month and then died.

In those cases that do have acute anemia, coming on rapidly after rupture, immediate operation should be done, even though the patient be absolutely pulseless, because you can never hope to accomplish anything by waiting. Fill them up with salt solution, go into the abdominal cavity, get hold of the bleeding point and stop it—then trust to luck.

Then, again, there is the other class of cases, and I have operated on several, where I had classical symptoms, and on going into the abdomen found no pregnancy. I have done that three or four times, and am quite willing to confess it. And I think that it has occurred to me about an equal number of times to go into the abdomen, thinking I had something else, and find a ruptured tubal pregnancy.

Dr. Charles A. L. Reed, of Cincinnati, O.—This subject is one about which it is difficult to keep silent when it is under discussion. I have been much interested, particularly in some diagnostic points which it raises. I shall not soon forget the dictum of Mr. Lawson Tait, as I used to hear him thunder it forth, that "it is impossible to diagnosticate ectopic pregnancy before rupture.'' Repeated experiences by numerous observers have broken the force of this dogmatic assertion, as most dogmatisms are more or less destroyed by the accumulated experience of the profession. I have myself made the diagnosis before rupture, and have operated before rupture; I have been called to operate in the practice of other people who have made the diagnosis before rupture, and have removed the specimen. Therefore the observations which I have made personally are only those which have occurred in the hands of other specialists, and in the hands of general practitioners, who are today the most acute students of the symptomatology of disease.

Now, this being true, I may be called upon in the light of the paper which has just been presented, and the remarks of my friend, Dr. Frederick, to define my practice, because I operated before there was a rupture, and not under menace of exsanguination and immediate death from bleeding; but I operated on the strength of the diagnosis, simply because I did not feel that it was well for the life of any woman to be walking around with a dynamite bomb in her, that may explode at any moment. That has been the key-note to my practice in these cases. I have never thought it safe to leave an ectopic pregnancy in situ, bleeding much or little, particularly if bleeding much; I have never thought it safe to leave it in position, *if it were there at all.* No woman has one minute's guarantee of life, much less of health, so long as she is the victim of this abnormal condition. I recollect the tragic case reported by my friend, Dr. Douglas, of Nashville, who was called to see a woman stricken at the counter in a drygoods shop, whom he found dead upon the floor. Shall we permit anybody to go about liable to a result so almost instantaneous, with a condition unattended to, which renders it possible? My own opinion, therefore is, that in the presence of pain and these eccentric signs of pregnancy, it is better first and foremost to examine your patient; study carefully the conditions that exist; and if ectopic pregnancy is found, then as speedily as the arrangements can be made relieve this woman of the sword of Damocles which hangs over her as long as the condition continues. I believe that to do less than this, is to do less than our duty to suffering womanhood that entrusts itself to our care. I am perfectly well aware that slight rupture and more or less hemorrhage may take place, and we may find concentric layers of clots that have been formed for a long time; within a week I have removed one of precisely that form, but because that woman had gone through three months of suffering and had not died, it becomes no argument that I should submit her or any other woman to a like danger. Therefore, I believe that the line of safe practice is to operate on an ectopic pregnancy whenever it can be diagnosticated. If we are to take any extreme position on this question, this certainly is the safer of the two. Expectant treatment is very likely to disappoint expectations.

Dr. Matthew D. Mann.—I have had considerable experience with ectopic gestation having seen perhaps a hundred cases; of these

I have seen only three where I thought it necessary to operate immediately. In these the hemorrhage was so great and the exigencies so severe that I operated within a few hours after seeing them.

I believe that the symptoms may be very much masked, and we may be led widely astray by looking out for classical symptoms. A couple of years ago I had four cases in two weeks; of the four I diagnosticated two, while the other two I didn't even suspect, but opened the abdomen looking for something serious, and found extrauterine pregnancy. I think everyone who does abdominal surgery must meet a good many where they suspect something else. One curious fact with regard to one of these cases impels me to relate it. Although she had an extrauterine pregnancy, she was normally pregnant at the same time, because seven months afterward she had a child which showed all the evidence of being full term, although an anencephalous monster. The woman is now pregnant again. I have seen two cases where extrauterine pregnancy occurred twice. I think there are a good many cases on record where it has occurred in the same woman twice.

We should all be on the look out for these cases and Dr. Reed has given us a striking illustration of the rapidity with which disaster may occur. I remember a case in Buffalo where the woman died suddenly and the husband was arrested for murder, supposed to have poisoned her.

Dr. C. C. Frederick.—I wish to state that the last case I operated on was a double-header, a gestation sac on each side.

